# Tempo is the key

**DOI:** 10.7554/eLife.63385

**Published:** 2020-11-03

**Authors:** Antonio Celani

**Affiliations:** Quantitative Life Sciences, International Centre for Theoretical PhysicsTriesteItaly

**Keywords:** olfaction, navigation, odor plumes, turbulence, olfactory search, wind tunnel, *D. melanogaster*

## Abstract

Walking flies find the source of attractive odors by changing how frequently they stop and turn in response to the smell.

**Related research article** Demir M, Kadakia N, Anderson HD, Clark DA, Emonet T. 2020. Walking *Drosophila* navigate complex plumes using stochastic decisions biased by the timing of odor encounters. *eLife* **9**:e57524. doi: 10.7554/eLife.57524

Tracking odors is a matter of life or death for most organisms, and insects are no exception (Baker et al., 2018). Male moths can track the pheromones of their potential mating partners from hundreds of meters away, and even fruit flies successfully engage in long-range searches for food using their sense of smell. However, this task is far from easy. This is because the molecules that carry the odorous message are transported by turbulent wind, where they get mixed with other molecules and form complex structures (Murlis et al., 1992; Crimaldi and Koseff, 2001). As a result, the information about the origin of a scent is seemingly lost, hidden in the intricacies of the intermittent odor signal. Insects appear to be able to overcome this problem, but it is unclear how they extract information about the origin of the odor and translate it into behaviors that allow them to find the source.

A major hurdle in the way of understanding how animals track smells is the need to visualize odors and behaviors simultaneously. While tracking insect behaviors in the wild remains a daunting task, wind-tunnel experiments provide a way to collect high-throughput data in controlled situations (Álvarez-Salvado et al., 2018). However, the artificial environment still poses several challenges: for example, it is unclear whether naturally occurring stimuli can be reproduced, or if it is possible to visualize odor concentrations with sufficient resolution.

Now, in eLife, Thierry Emonet and co-workers from Yale University – including Mahmut Demir and Nirag Kadakia as first authors – report how the fruit fly *Drosophila melanogaster* behaves in response to an attractive smell while walking (Demir et al., 2020). Quite serendipitously, they discovered that starved flies are attracted to smoke, which can be easily visualized. By manipulating the airflow in a wind tunnel with lateral jets, they generated a stream of smoke with properties similar to the odor signals that flies encounter in the wild (Celani et al., 2014). In agreement with theoretical expectations, they found that, within the smoke, flies have brief, frequent and unpredictable encounters with the odor. But how do these encounters modulate flies’ behavior?

Demir et al. observed that the rich variety of movements exhibited by walking *Drosophila* could be summarized into just a few behavioral states relevant to olfactory navigation, echoing previous findings (Tao et al., 2019). They found the search process was inherently stochastic: periods of walking in a straight line would randomly be interrupted by rapid turning events or by stopping for longer extents of time.

By comparing the trajectories recorded with or without smoke, but always in presence of turbulent airflow, Demir et al. were able to identify which features of the flies’ movements were affected by encountering the smell. They found that the walking pace, the frequency and speed at which the flies made a turn, and the sharpness of the turns were not affected by the smoke. Conversely, when the smoke was present walks were on average longer and stops shorter. Most importantly, when the flies were turning, they were more likely to reorient upwind against the direction of the wind if they had already encountered the odor. But what are the specific characteristics of the odor that the fly perceives and responds to?

Demir et al. found that neither the concentration of the odor nor the amount of times flies were exposed to it played a significant role in the search process. Instead, the tempo of the flies’ encounters with the odor appeared to be the key determinant of the decision-making process. In a stopped fly, a single encounter was sufficient to initiate a walk, and several encounters close together shortened the duration of the stops. Walking times increased after an encounter, but further exposure to the odor shortly after did not lead to a cumulative effect. This modulation of walks and stops produces a bias that results in the fly visiting regions of space where it is more likely to encounter the smell. Further experiments showed that above a certain frequency of encounters flies were more likely to re-orientate themselves upwind. In fact, the combined effect of the odor on the frequency of walks and stops, as well as the direction of the turns, proved to be fundamental for the flies to find the origin of a smell (Figure 1).

**Figure 1.**
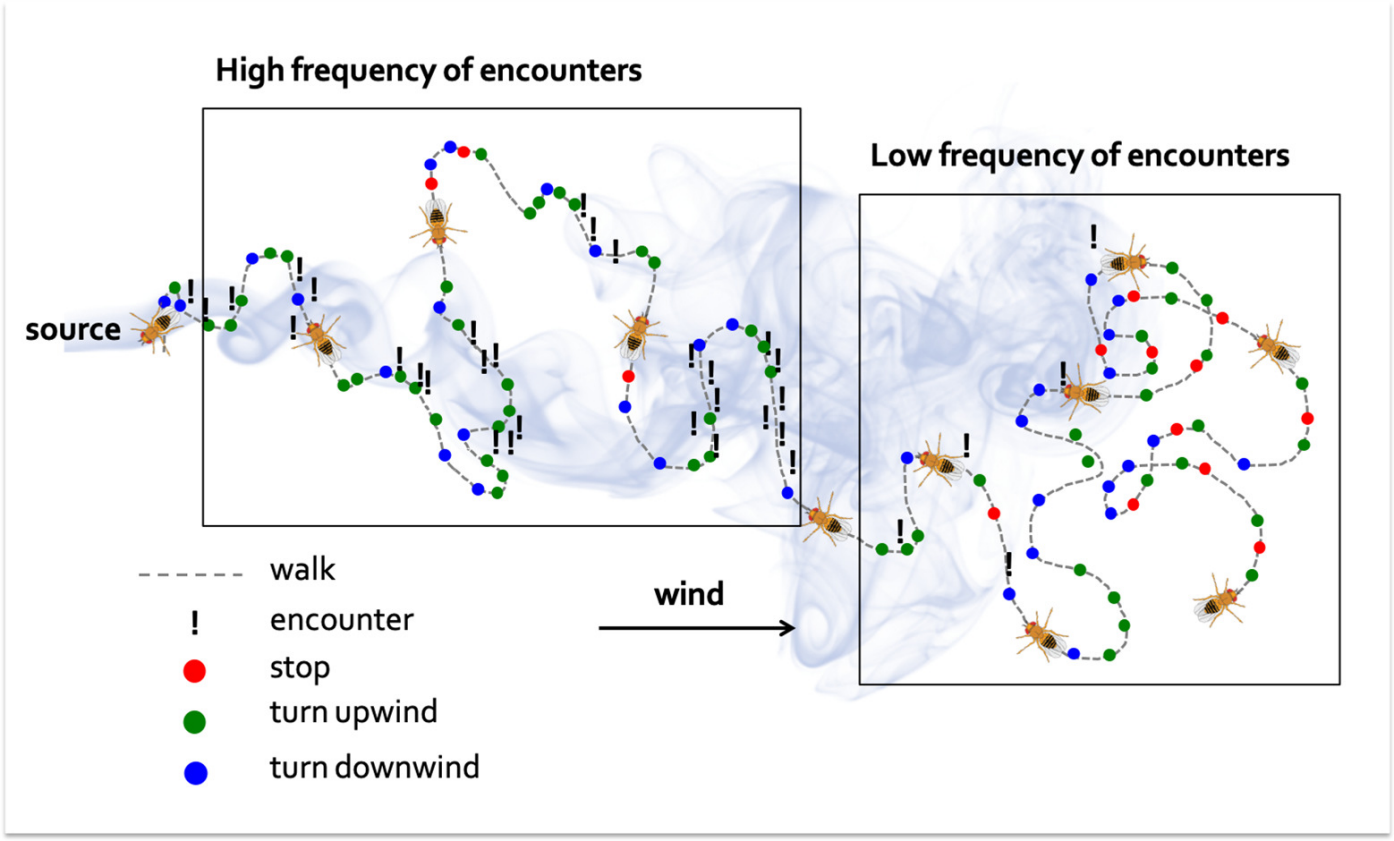
How a walking fly navigates towards the source of an odor. Flies that have been starved are attracted to smoke. In this schematic diagram of a wind tunnel experiment, the fly starts on the right, the source of the smoke is on the left, and the wind blows from left to right (black arrow). When far from the source (right box), the fly is less likely to encounter the odor, leading to frequent stops (red dots) and a similar number of upwind and downwind (green and blue dots) turns. Once the fly gets closer to the source (left box), it is more likely to encounter the odor, and the number of stops decrease, while the frequency of upwind turns increases.

There are tantalizing similarities between the olfactory-search strategies of walking flies and other insects, but also significant differences (Mafra-Neto and Cardé, 1994; Budick and Dickinson, 2006). In any event, irrespective of their sizes and behaviors, different species must overcome the same challenges posed by the transport of odor molecules by the turbulent airflow, pointing to the existence of general underlying principles. Perhaps, in the future, more studies like this one will pave the way towards a more fundamental understanding of long-range olfactory navigation.
